# Response analysis of orthotropic steel deck pavement based on interlayer contact bonding condition

**DOI:** 10.1038/s41598-021-03137-7

**Published:** 2021-12-08

**Authors:** Xuntao Wang, Changhao Zhang, Ruijuan Sun

**Affiliations:** 1 Xi’an Shiyou University, Xi’an, 710065 China; 2 Jinan City Planning and Design Institute, Jinan, 250101 China

**Keywords:** Civil engineering, Mechanical engineering

## Abstract

In this research the interlayer contact condition was considered between the adjacent layers of orthotropic steel deck pavement, and an interface contact bonding model was applied to simulate the interlayer bonding condition and evaluate the response of deck pavement under vehicle loads. An advantage of this model is that it can simulate not only the full-bond condition but also the debonding condition at somewhere between adjacent layers. The responses of the orthotropic steel deck pavement were calculated and analyzed by the model, and it found that this model is reasonable and credible to evaluate the responses of the deck pavement comparing with the previous researches. The full-bond condition was an ideal condition between adjacent layers, which was prone to underestimate the responses and deformation of the deck pavement. Moreover, the position and size of the disengaging area have a notable influence on the tensile strain at the top of SMA layer and the bottom of GA layer, and the tensile strain of them also increase with the increase of the disengaging area. Finally, the responses of the steel deck pavement changed obviously when the vehicle speed increase, so the suitable speed limit may reduce the responses and deformation for prolonging the service life of the orthotropic steel deck pavement.

## Introduction

Orthotropic steel deck (OSD) has the characteristics of high strength-to-weight ratio, ease of assembly, and modular construction, and therefore widely used in the construction of long-span bridges and urban bridges^1^. Compared with concrete bridges, the high degree of pre-fabrication, lightweight-design, and rapid erection capability of this type of steel bridges make it a better option for many applications, including long-span bridges, movable bridges, and bridges with restricted beam depth^2^. Asphalt concrete deck pavement is an important role paved on OSD bridges, which is used to provide the essential functions of load distribution and skid resistance, and to protect the steel bridge deck as well as providing service to the vehicle^3,4^. The asphalt concrete deck pavement is exposed chronically and directly to adverse environmental condition and traffic conditions, and this reduce its service life compare with other parts of the OSD bridges^5^. Therefore, the asphalt concrete deck pavement needs to be designed carefully to provide the high service level and the long service life of the OSD bridges^6^.

Asphalt mixtures are the mainly and commonly paving materials which are used for building the OSD pavement, such as gussasphalt (GA), stone mastic asphalt (SMA), and epoxy asphalt (EA) mixtures^7–10^. However, the mechanical behaviors of these asphalt mixtures change much significant with the climate, and also affected by vehicle speed^11^. Therefore, the responses of OSD pavement should be calculated and analyzed under different temperature and loading conditions in order to make a suitable evaluation and obtain a deep understanding of the mechanical properties of OSD pavement. Besides, the bonding condition between the adjacent layers is an important factor, which affects the mechanical responses of OSD pavement in finite element simulation^5,6,12^. The good bonding condition between adjacent layers can help the asphalt concrete surface course and the steel deck pavement form an integral structure to resist the external loads, on the contrary, the weak bonding between adjacent layer is disadvantage for steel deck pavement and prone to cause deck pavement diseases. In some of previous researches^4,6,8,13–16^, full-bond condition and no-bond condition are two commonly bonding condition between adjacent layers, however, a few researchers hold that the interlayer bonding condition of adjacent layers lies in somewhere between full-bond condition and no-bond condition^5,11,12,17^. In a recent study^18^, the ultra-high performance concrete (UHPC) has been utilized to reinforce the orthotropic steel bridge, which is an innovative cement-based material for enhancing the stiffness of bridge deck and improving the fatigue performance. The composite action between UHPC layer and the deck plate of OSD were realized by shear studs and coupling the vertical translational degrees of freedom, i.e., partial slip. So the interlayer condition need be considered in simulating the bonding condition between adjacent layers of OSD pavement.

In this study, an orthotropic steel bridge with two-layer paving structure was investigated to estimate the responses and performance of the deck pavement. The first objective is to build a finite element model that can simulate the interlayer bonding condition between adjacent layers of the OSD pavement accurately. To accomplish the objective, the interface contact bonding model was built to evaluate the response of orthotropic steel deck pavement under vehicle loads, where the interface condition is different from full-bond condition and no-bond condition. The model can simulate not only the interface bonding condition between adjacent layers but also the phenomenon of bonding failure at somewhere between adjacent layers, which is the obvious difference with the model used by the other related researches. The second objective is to predict the responses of OSD pavement by the developing model under the different temperature and loading conditions. The temperature has an effect on the performance of the asphalt mixtures, which makes the deformation of the OSD bridges with asphalt pavement notable at the high temperature. So the responses of OSD pavement will be calculated and analyzed by the interface contact bonding model under the high temperature to understand the mechanical behaviors of the deck pavement. Meanwhile, the effects of bonding failure on the response of the OSD pavement will be analyzed and discussed, and it is helpful for knowing the bonding failure between adjacent layers to the damage of deck pavement. Moreover, the influence of the thickness of the deck plate and the U-rib on the responses of deck pavement was conducted respectively. Finally, it is expected that the findings in this research will help to obtain a better insight into the responses of the OSD pavement.

## Methodology

### Steel deck pavement structure and traffic loading

**Figure 1:**
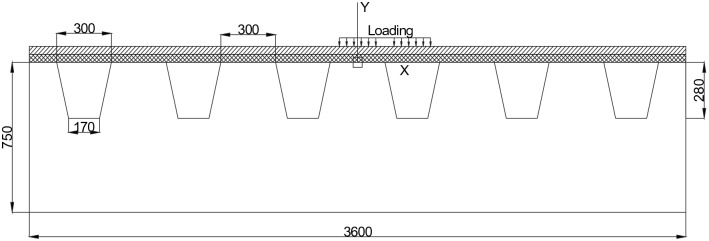
The section of OSD pavement structure (Unit: mm, not to scale).

A three-dimensional finite element model with ‘deck-pavement’ was built by ANSYS software, and the model is a simplified structure model for simulating and calculating the responses of the OSD pavement. This structure model is employed by some researchers in their studies^5,6,8^, and it is proved to be reasonable for evaluating the responses of the OSD pavement. Otherwise, this type of model has the general characteristics of OSD pavement structure, which has the universality, and it can also reduce the time for computing. In this paper, the section of the OSD pavement structure is shown in Fig. 1, and it consists of beam plates, deck plate, U-rib stiffeners and two surfacing layers (GA layer and SMA layer). There are six U-ribs for supporting the steel deck plate in longitudinal direction (Z axis direction, same as the traffic direction), and there are four cross beams for supporting the steel deck plate in transverse direction (X axis direction). The thickness, width and length of the steel deck plate are 14 mm^19^, 3600 mm and 9000 mm, respectively. The thickness and depth of the cross beam plate are 10 mm and 750 mm, respectively, and the spacing between two adjacent cross beams is 3000 mm. The thickness of U-rib is 8 mm. The vertical pressure applied on the surface of the pavement layer was used to simulate the traffic loading, which is corresponding to a single axle with dual-tires, and placed at the right of the midspan.

**Figure 2:**
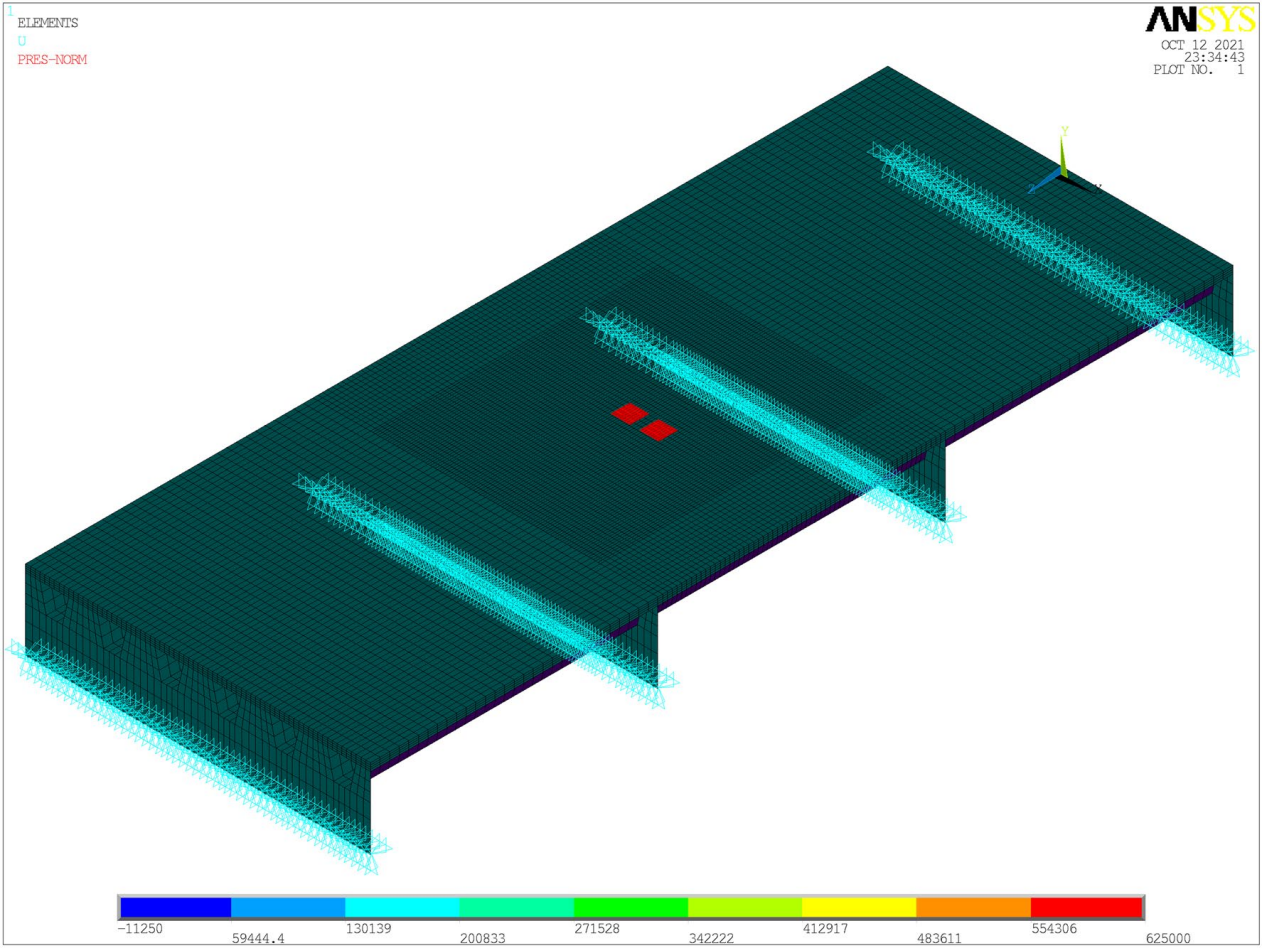
The EF model of OSD pavement structure (Unit: Pa).

As seen in Fig. 2, a relatively coarse mesh is used for the regions which are far from the loading area, and a relatively fine mesh is used for the regions which are around the loading area. The element thickness of 1 cm was used for the GA layer and the SMA layer. There are 80,640 solid elements which are used for the deck pavement and 24,480 shell elements which are used for the deck plate, U-ribs and cross beams after the ‘deck-pavement’ structure are meshed. The contact area of each tire is 400 cm^2^, which is a 20 cm × 20 cm rectangular loading area and red regions shown in Fig. 2, and the space between two tires is 10 cm^1,20^. The contact pressure corresponding to the traffic loading was 0.625 MPa.

The force of rolling friction $f$ between the surface of steel deck pavement and the tires is determined by Eq. (1):

1 $$f = \mu P$$

where $\mu$ is the coefficient of rolling friction, and in this study it is 0.018. $P$ represents pressure. As for the boundary conditions of the finite element model, the bottom of the beam plates were fixed in three directions, and the other peripheries are fully free.

### Material characterization

**Figure 3:**
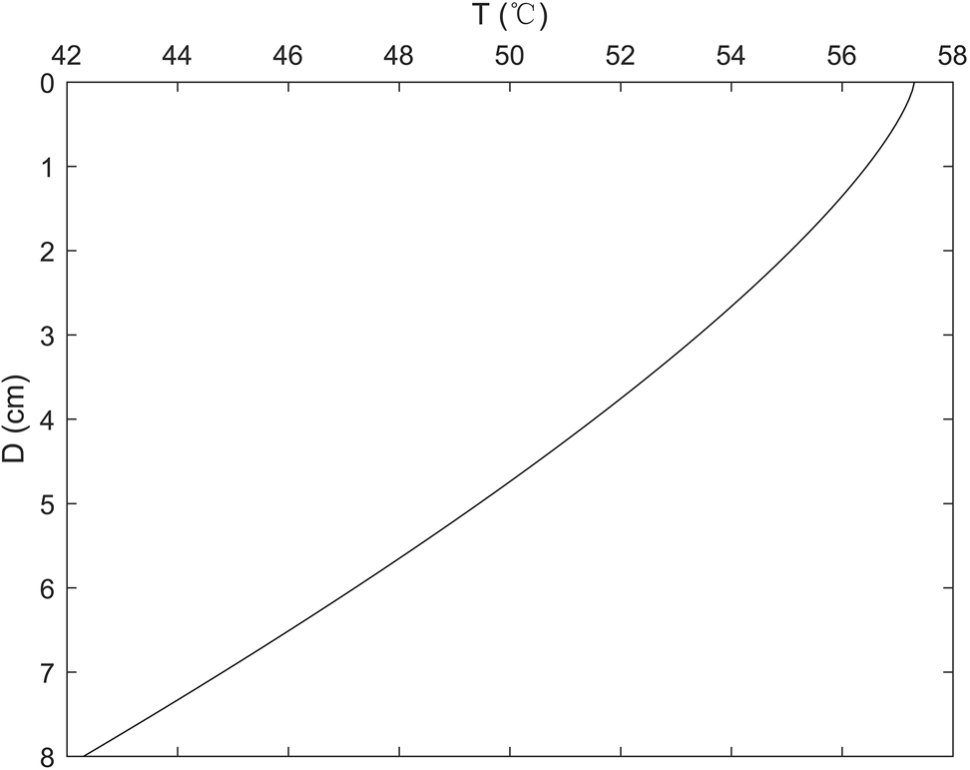
Temperature profile along the depth of deck pavement.

The materials are the SMA and GA for the deck pavement and steel for the components of the orthotropic steel deck. It is well known that the SMA used for the surface layer has an excellent rut-resistance and fatigue cracking resistance. The GA used for the base course bonds quite well to orthotropic steel deck, and the impermeability of it is also very well. However, the responses of these asphalt mixture are affected by environment temperature and loading time dramatically. The deck pavement is prone to deform in the high temperature compare with the normal temperature, then it may turn into damage under traffic loading. Therefore it is more valuable to calculate and analyze the responses of the deck pavement under the high temperature. It should be noted that the temperature of each layer of the deck pavement is varying with the depth and environment, and then in this study the temperature of the deck pavement was calculated by the following formula (In Summer)^21^:

2 $$T = - 0.86D^{1.375} + 0.742T_{a\max } + 1.334R_{0} - 0.0542H_{ave} - 0.0693W_{ave} - 24.509$$

where $T$ is the temperature (℃), $D$ is the depth (cm), which is measured from the pavement surface, $T_{a\max }$ is the maximum temperature in a day (℃), $H_{ave}$ is the average of the humidity in a day (%), $W_{ave}$ is the average of the wind speed in a day (km/h), and $R_{0}$ is the solar radiation in a day, which is calculated as follow:

3 $$R_{0} = 41{\text{.8632}}E_{0} \sin (\varphi )\sin \left( {\delta \left( {\frac{\pi \omega }{{180}} - \tan (\omega )} \right)} \right)$$

4 $$E_{0} = 1{{.00011 }}- 0{\text{.034221cos(}}\Gamma {) + 0}{\text{.00128sin(}}\Gamma {) + 0}{\text{.000719cos(2}}\Gamma {) + 0}{\text{.000077sin(2}}\Gamma {)}$$

5 $$\begin{gathered} \delta = \frac{180}{\pi }{(0}{\text{.006918}} - 0{\text{.399912cos(}}\Gamma {) + 0}{\text{.070257sin(}}\Gamma {) - 0}{\text{.006758cos(2}}\Gamma {)} \hfill \\ \quad{ + 0}{\text{.000907sin(2}}\Gamma {) - 0}{\text{.002697cos(3}}\Gamma {) + 0}{\text{.00148sin(3}}\Gamma {))} \hfill \\ \end{gathered}$$

6 $$\omega = \cos^{ - 1} ( - \tan (\varphi )\tan (\delta ))$$

7 $$\Gamma { = }{{2\pi (dn - 1)} \mathord{\left/ {\vphantom {{2\pi (dn - 1)} {365}}} \right. \kern-\nulldelimiterspace} {365}}$$

where $\varphi$ is the value of latitude (°), which means the latitude of observing site, $dn$ is the serial number of date in a year, which is in the range of 1 to 365, such as January 1st, *dn* = 1, and July 1st, *dn* = 181. In this paper, Xi’an (in China) is the observing site, and the value of the latitude is about 34 (°). *dn* = 187, $T_{a\max }$ = 39 (℃), $H_{ave} = 25$(%),$W_{ave} =3.6$ (km/h), then the temperature profile of deck pavement according to above formulas and parameters was shown in Fig. 3. The temperature of the orthotropic steel bridge is assumed to be unchanged and keep a stable state. Furthermore, the material properties of the OSD pavement and the thickness of asphalt concrete are listed in Table 1 at different temperature, and the temperature of GA layer and SMA layer at high temperature are expressed by the temperature at the center of its thickness, respectively.

**Table 1:** Material properties of the steel deck and pavement.

Material	Normal temperature		High temperature		Poisson’s ratio	Density (kg/m^3^)	Thickness (cm)
	Temperature (°C)	Modulus (GPa)	Temperature (°C)	Modulus (GPa)			
SMA	20	3.255	55 $(D = 2)$	0.194	0.35	2300	4
GA	20	4.968	47 $(D = 6)$	1.415	0.35	2400	4
Steel	–	210	–	210	0.25	7850	–

Moduli of the GA and SMA were determined at 10 Hz.

### Interface contact bonding model

The full-bond condition between adjacent layers, especially the deck plate and the pavement layer is a desired result in the ‘deck-pavement’ structure. The good bonding condition between adjacent interfaces will help the ‘deck-pavement’ structure constitute a monolithic structure to resist against the effect of traffic and environment^11^. On the contrary, the poor bonding condition will reduce the shear strength between adjacent interfaces, thereby decrease the capability for stress transferring and energy dissipation and lead to several pavement distresses. The full-bond or no-bond condition is an ideal interface bonding condition, which were usually used to predict the responses of the pavement structure and steel deck pavement in some of previous researches. However, in recent years some researches and early diseases of pavement show that the interface bonding condition of adjacent layers are neither fully bond (full-bond condition) nor completely debond (no-bond condition) with each other, but lies in somewhere between these two ideal interface bonding condition.

**Figure 4:**
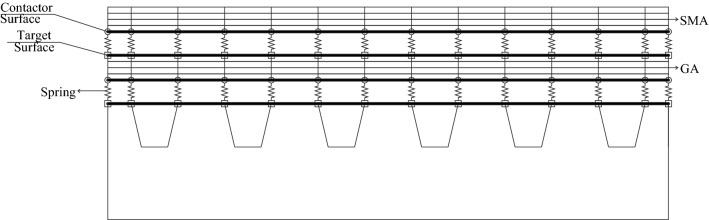
Schematic of interlayer contact bonding model (Not to scale).

In this study the interface condition of adjacent layers are supposed to be interface contact bonding condition (ICB condition), and the Fig. 4 displays the concept of interface contact bonding model (ICB model)^11,12^. In the ICB model, the upper layer and lower layer of the adjacent layers are covered by contact element and target element respectively, and contact element and target element are connected by three-direction translational spring element at the corresponding nodes. Then two kind of contact bonding interfaces are built, which are applied to imitate the interface condition between the SMA layer and the GA layer and the interface condition between the GA layer and the deck plate, respectively. The translational springs between contact element and target element can measure the value of compression or tension in each direction, and the stress and strain between adjacent layers will be calculated by the measured value according to contact algorithm^22^. $K_{n}$ is a coefficient of translational spring, which is normal stiffness of the spring along y direction. $K_{t}$ and $K_{s}$ are coefficients of translational spring too, which are tangent stiffness of the spring at *x* direction and *z* direction, respectively. Once the material parameters are determined and the mesh is completed, the three stiffness coefficients can be generated automatically based on the contact algorithm. But sometimes a reduction coefficient may be used to modify the three stiffness coefficients to get a better computation.

The stress along normal and tangent direction at the adjacent interface will be computed according to the augmented Lagrangian method^22^ under the ICB condition, and the tangent stress between the adjacent layers satisfies the following conditions^11^:

8 $$\tau_{\lim } = \mu P + C$$

9 $$\left| \tau \right| \le \tau_{\lim }$$

In Eq. (8), $\tau_{\lim }$ is ultimate stress along tangent direction between adjacent layers. $\mu$ is the coefficient of sliding friction between adjacent layers.$P$ is stress of compression or tension in normal direction.$C$ is bonding stress at adjacent layers. In Eq. (9), $\tau$ is equivalent stress along tangent direction between adjacent layers. Now let's analyze the deep meaning of the Eqs. (8) and (9). In Eq. (8), assuming that the stress $P$ is zero and the bonding stress $C$ at adjacent layers is not zero, then the adjacent layers bond together because of the bonding stress. On the contrary, the phenomenon of bonding failure will appear between adjacent layers as the bonding stress $C$ is none. In Eq. (9), if the equivalent stress $\tau$ at adjacent layers is less than or equal ultimate stress $\tau_{\lim }$, the corresponding nodes at the adjacent layers keep sticking state, otherwise the corresponding nodes at the adjacent layers come into slide. In this study, the bonding stress between the adjacent interfaces is 0.5 MP^12^, and $\mu$ is 0.5.

**Figure 5:**
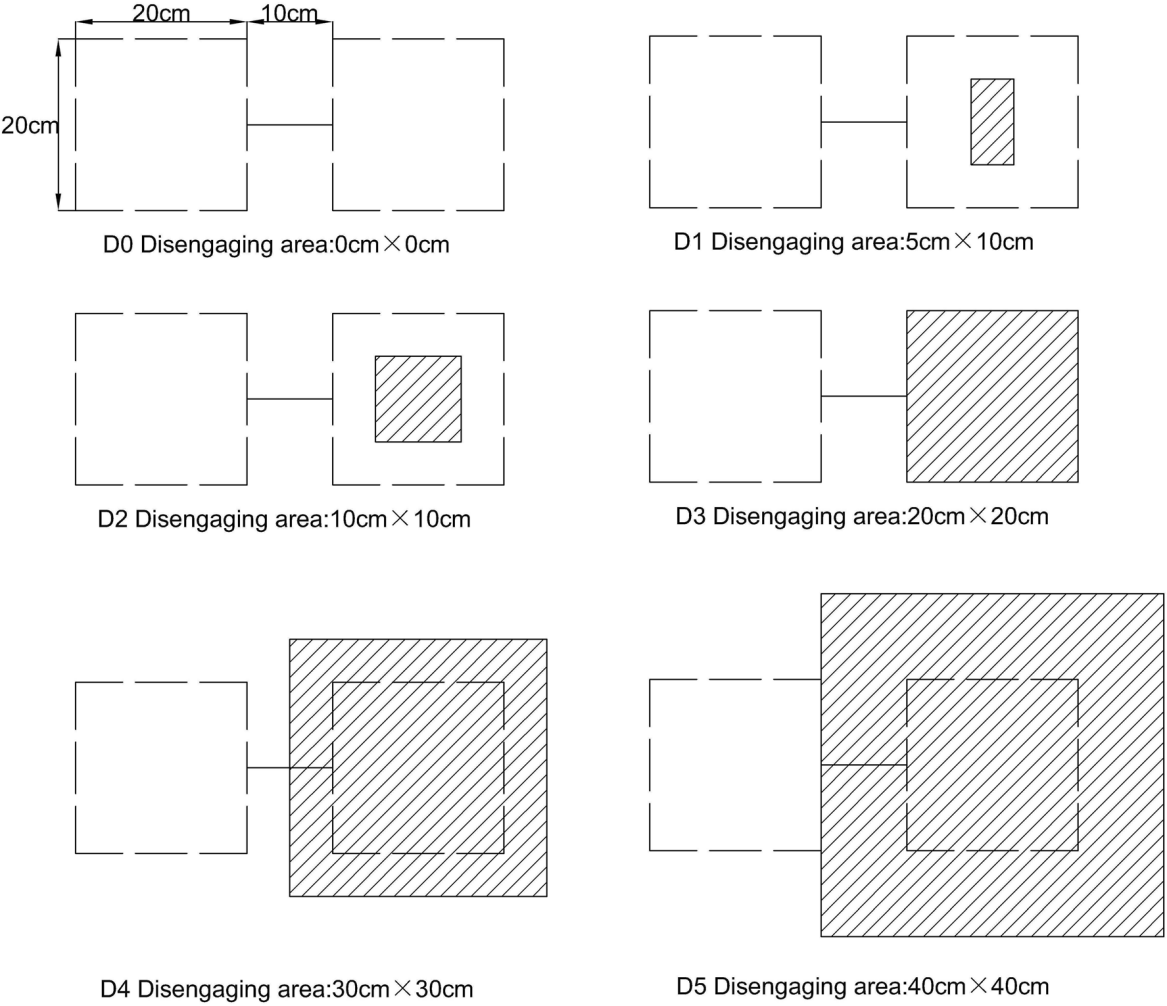
Type of disengaging area under loading area (Unit: cm^2^).

The phenomenon of insufficient bonding strength or bonding failure between adjacent layers at somewhere may not be simulated by previous model, but these phenomena do occur sometime during the service life of pavements. The ICB model can easily capture these phenomena by adjusting the value of the bonding stress $C$ of contact element. When the bonding stress between adjacent layers at somewhere is assumed to be 0 and keeps unchanged (not zero) elsewhere, the ICB model can be used to simulate the phenomenon of bonding failure at local place. In this study, it is assumed that the phenomenon of the bonding failure take place between the GA layer and the deck plate at somewhere, and the disengaging area is a rectangle. Six types of disengaging area are listed and shown in Fig. 5, and used to imitate the phenomenon of bonding failure at local place between the GA layer and the deck plate. In Fig. 5, the area within the dashed box is the area of dual wheel loading on the top of the SMA layer (Vertical view), and the disengaging area between the GA layer and the deck plate is the shaded area under the loading area. In type “D0”, there is no the shaded area, and this means that the GA layer and the deck plate have no disengaging area under the loading area. Furthermore, in other types none of the shaded area between the GA layer and the deck plate is zero, which means that there are the disengaging area under the loading area, and the size of the disengaging area increase from type “D1” to type “D5”. In type “D3”, the disengaging area was 20 cm × 20 cm, and this represents that the disengaging area between the GA layer and the deck plate is same as the loading area of one wheel. In type “D5”, the disengaging area between the GA layer and the deck plate was 40 cm × 40 cm, and this indicates that the size of the disengaging area is four times as much as the size of the loading area of one wheel.

### Comparison of result with other models

**Figure 6:**
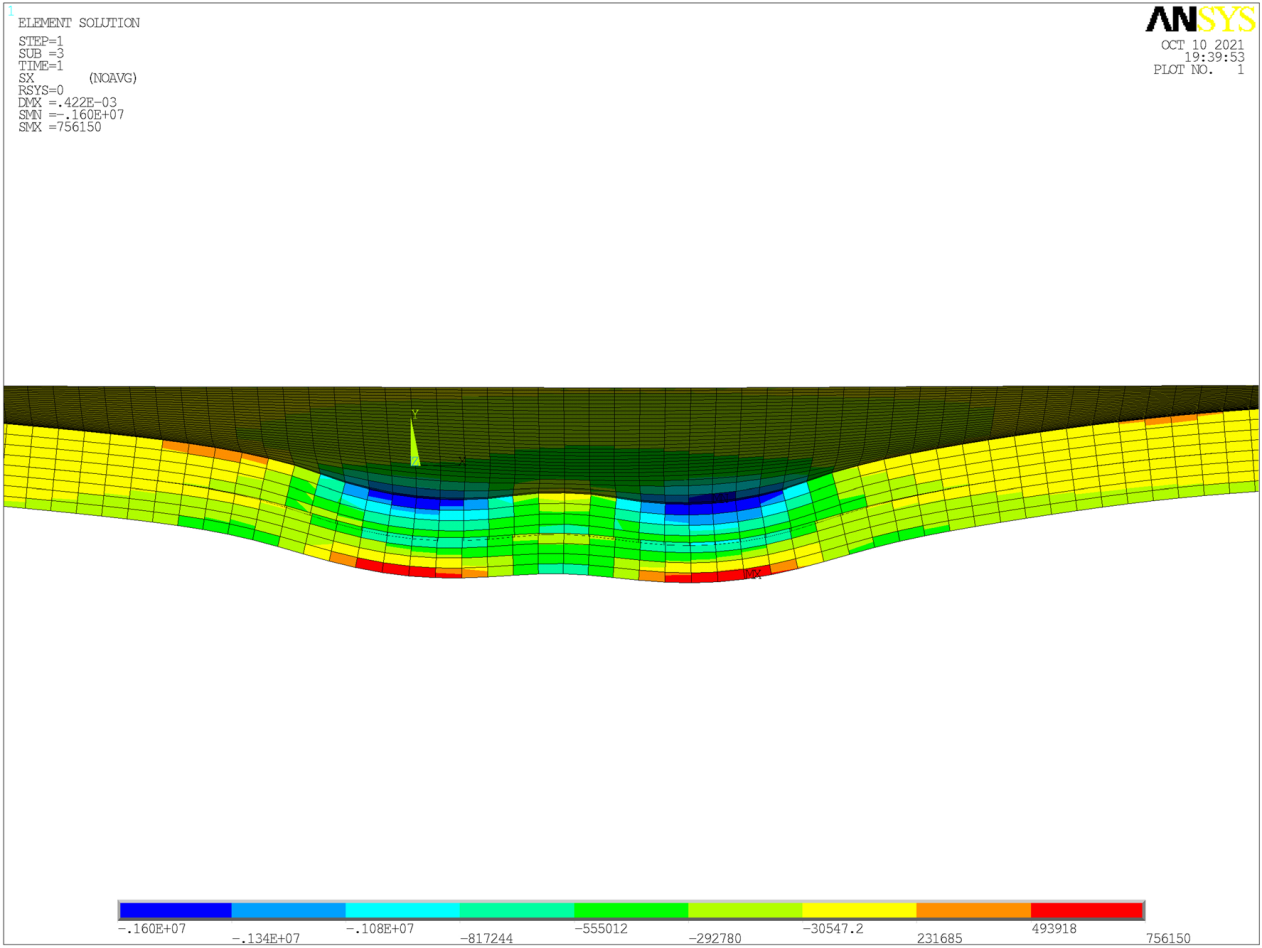
Transverse stress distribution in the deck pavement (Unit: Pa).

In order to estimate the accuracy and rationality of the ICB model, the response of the deck pavement was calculated under static loads and moving loads at the normal temperature and compare with the result of other researches. For validating our model, we find that in the literatures^5,6^ the geometric parameters of the OSD bridge with asphalt pavement and the loading position are almost at the same with them in ours, so these researches are chosen to validate our model. However, the notable difference between the literatures^5,6^ and our study is the interface condition of adjacent layers. In the literatures^5,6^, the interface condition between the adjacent layers is the full-bond condition, but the interface contact bonding condition was used in this research. The transverse stress of the deck pavement under static loads is shown in Fig. 6, and the region of tension and compression keep consistent with the studies by Kim et al.^5^. The results from Kim et al. are verified by a strain gauge and a linear variable differential transducer, and the responses of the transverse stress of the deck pavement are similar to other studies mentioned in their research. Tensile stress, which was marked red in Fig. 6, occurred at the top of the deck pavement, which is near the loading, as well as the bottom of the deck pavement, which is below the loading. Compressive stress, which was marked blue in Fig. 6, occurred at the top of the deck pavement, which is under the loading.

**Figure 7:**
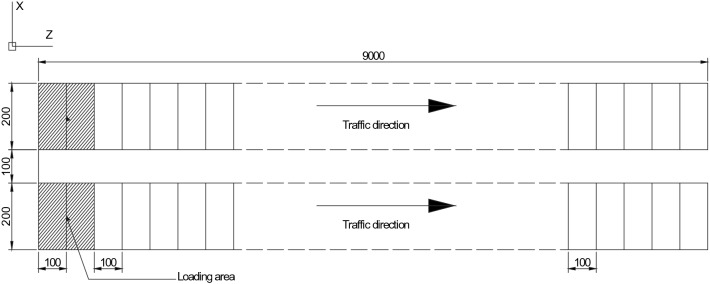
Schematic of moving loads (Unit: mm).

The responses of the deck pavement under the moving loads are calculated according to the following dynamic algorithm^1,23^:

10 $$[M]\left\{ {\ddot{u}} \right\} + [C]\left\{ {\dot{u}} \right\} + [K]\left\{ u \right\} = \left\{ F \right\}$$

where $[M]$, $[C]$, and $[K]$ = mass matrices, damping matrices, and stiffness matrices, respectively; $\left\{ {\ddot{u}} \right\}$, $\left\{ {\dot{u}} \right\}$, $\left\{ u \right\}$  = acceleration vector, velocity vector, and displacement vector, respectively; and $\left\{ F \right\}$ is the external force vector. In this study, the damping $C$ is assumed to be $\alpha_{1} M + \alpha_{2} K$, where $\alpha_{1}$ and $\alpha_{2}$ are Rayleigh damping coefficients. The responses of the deck pavement under different speed were calculated to analysis the behaviors of the deck pavement. In Fig. 7 it shows the schematic of moving loads, where the size of the contact area of the dual wheels is 400 mm by 200 mm, and the space of two tires is 100 mm. The total length of moving loads path is 9000 mm. There are totally 90 iterations, and in each iteration the vehicle moves forward 100 mm along the traffic direction. The responses of the deck pavement were computed at the normal temperature when vehicle pass through the deck pavement at 2 km/h, 10 km/h, 20 km/h and 40 km/h, respectively.

**Figure 8:**
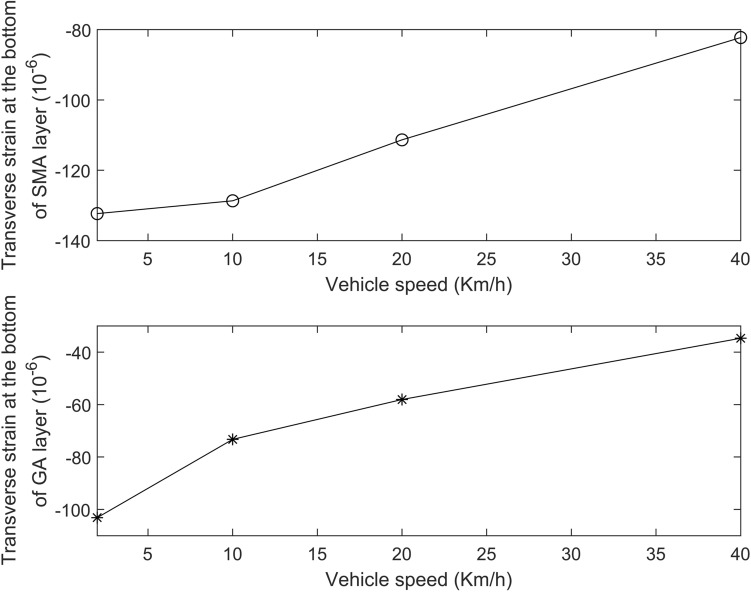
Transverse strain of the deck pavement under different vehicle speed.

The transverse strain at the bottom of SMA layer and at the bottom of GA layer was calculated between the dual tires, which are shown in Fig. 8. The trend of transverse strain of the deck pavement changes in a similar way with results measured by Cheng et al.^6^, who got the transverse strain of the OSD bridge with asphalt pavement under moving loads by strain measurements, and the magnitude and trend of transverse strain of the OSD pavement under different vehicle speed simulated by ours is in accord with the results of Cheng et al. Meanwhile, the transverse strain of the deck pavement all decease when the vehicle speed increase, this is same with the results of other researches^11,12^ including results of Cheng et al.

In summary, compared with the responses calculated or measured by other studies to verify the EF model we used, and the responses of the deck pavement we simulate basically stay in step with them in the literatures under the static loads or moving loads at the normal temperature. So the responses of the deck pavement calculated by the ICB model are basically reasonable and reliable.

## Critical response analysis of the deck pavement

The critical responses of the deck pavement play a crucial part in the structure design and materials requirement of pavement, especially at the high temperature. In order to analyze the influence of interface bonding condition and vehicle speed on the performance and service life of the deck pavement at the high temperature, the critical responses of the deck pavement would be calculated to study the strain and deformation of the deck pavement.

### Responses of OSD pavement with different interface condition

**Figure 9:**
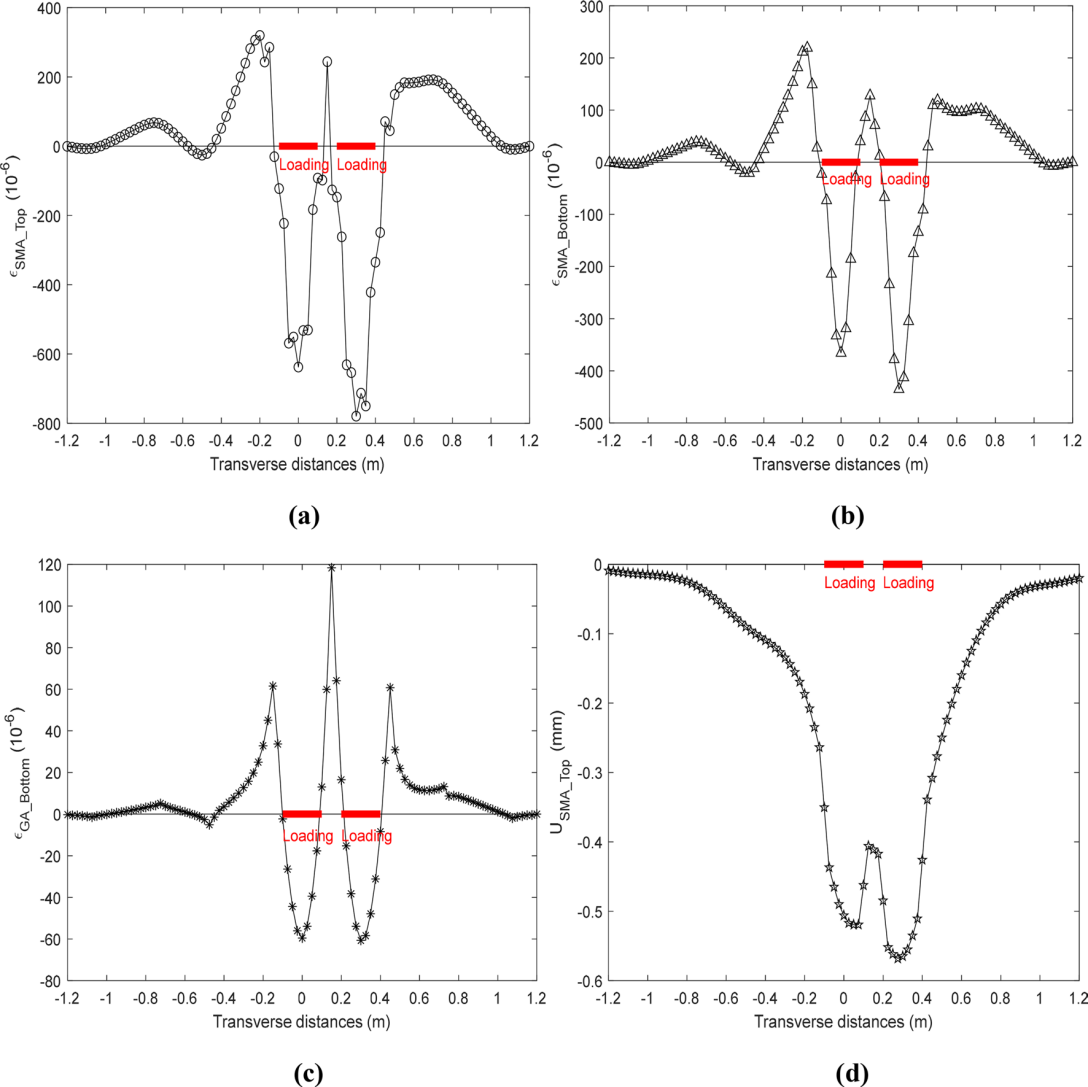
Response of deck pavement with full-bond condition.

The responses of the SMA layer and the GA layer at the top or bottom were computed, ranging from − 1.2 to 1.2 m along the X axis under dual-tires, where 97 nodes were defined in the response profile. The responses of the deck pavement with the full-bond condition are shown in Fig. 9 at the high temperature, where the loading area are marked red for presenting the loads of the dual-tires. The transverse strain at the top and bottom of the SMA layer (${\varepsilon _{{\text{SMA}}\_{\text{Top}}}}$ and ${\varepsilon _{{\text{SMA}}\_{\text{Bottom}}}}$) are shown in Fig. 9a and in Fig. 9b, respectively. In addition, the transverse strain at the bottom of the GA layer (${\varepsilon _{{\text{GA}}\_{\text{Bottom}}}}$) is shown in Fig. 9c, and the vertical displacement at the bottom of the SMA layer (${\text{U}}_{{{\text{SMA}\_\text{Top}}}}$) is shown in Fig. 9d. It’s easy to see that the strain response of the SMA layer and the GA layer are all in compression under the dual tires and in tension near the loading from the Fig. 9. The tensile strains of the SMA layer, which located on the left side of the loading area, are greater than it on the right side of the loading area, but the compressive strains at the top of the SMA layer are on the contrary. The tensile strains of the GA layer at the both side of the loading area almost are in same, and the vertical displacement at the top of the SMA layer, which located under the right side of the tire, is greater than it under the left side of the tire. Besides, the transverse strains of the SMA layer and the GA layer between the dual-tire are all in tension.

**Figure 10:**
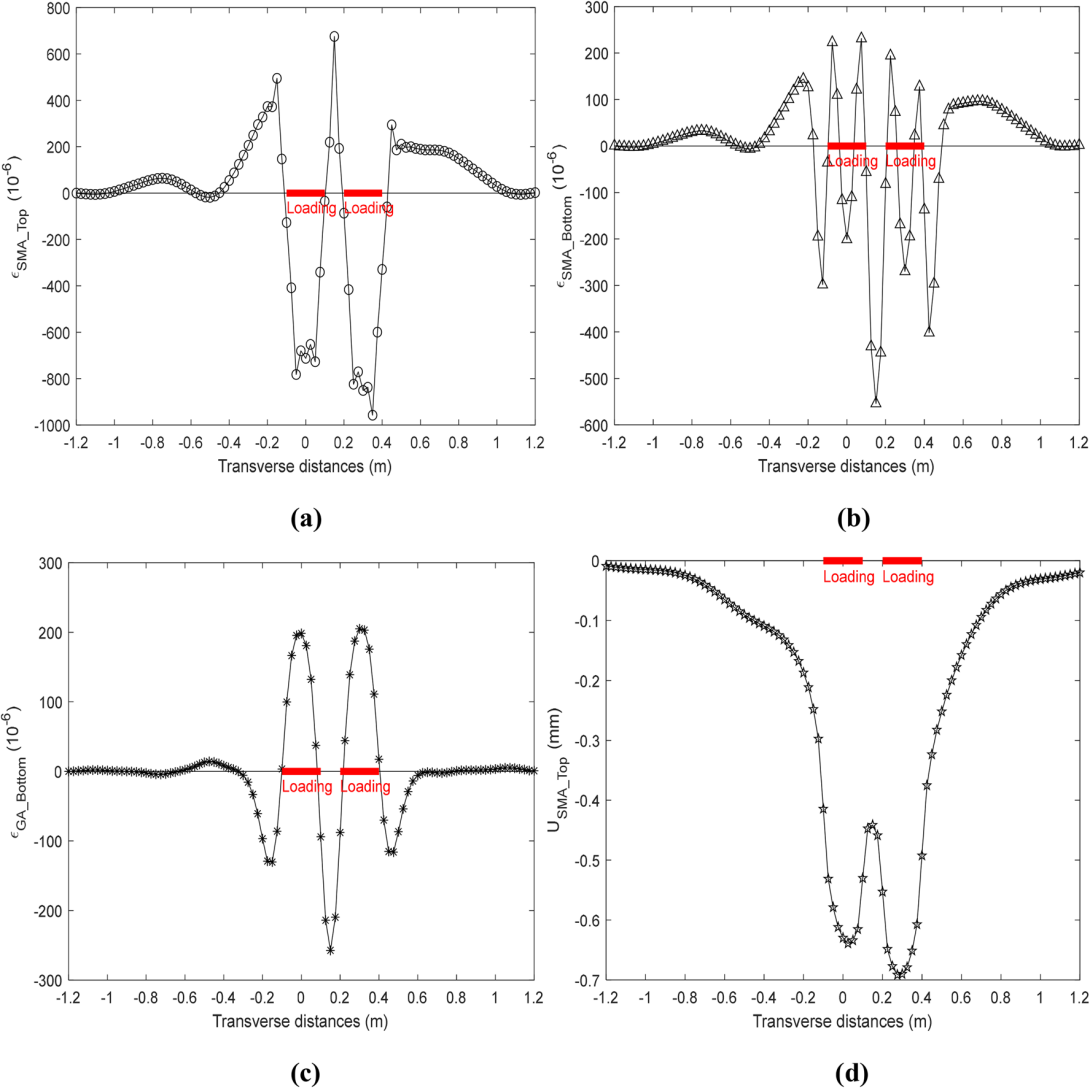
Response of deck pavement with ICB condition.

The responses of the deck pavement with the ICB condition are shown in Fig. 10 at the high temperature, where the transverse strain at the top and bottom of the SMA layer ($\varepsilon_{{{\text{SMA}\_\text{Top}}}}$ and $\varepsilon_{{{\text{SMA}\_\text{Bottom}}}}$), the transverse strain at bottom of the GA layer ($\varepsilon_{{{\text{GA}\_\text{Bottom}}}}$), and the vertical displacement at the top of the SMA layer (${\text{U}}_{{{\text{SMA}\_\text{Top}}}}$) are presented in (a), (b), (c) and (d), respectively. The $\varepsilon_{{{\text{SMA}\_\text{Top}}}}$ and the ${\text{U}}_{{{\text{SMA}\_\text{Top}}}}$ with the ICB condition have broadly similar trend to them at the full-bond condition, but the $\varepsilon_{{{\text{SMA}\_\text{Top}}}}$ and the ${\text{U}}_{{{\text{SMA}\_\text{Top}}}}$ under the ICB condition are all greater than them under the full-bond condition. The $\varepsilon_{{{\text{SMA}\_\text{Bottom}}}}$ X with the ICB condition change very sharply near the loading area, which is different from it with the full-bond condition. The $\varepsilon_{{{\text{GA}\_\text{Bottom}}}}$ in Fig. 9c almost have a converse trend with it in Fig. 10c, where the tensile strain and the compressive strain are just upside down, however, the $\varepsilon_{{{\text{GA}\_\text{Bottom}}}}$ with the ICB condition is more suitable than it with the full-bond condition according the results of previous researches. Besides, it’s easy to found that the maximum of the transverse tensile strain appear at the top of the SMA layer between dual tires or at the bottom of the GA layer at the ICB condition.

In this study, ${\text{U}}_{MST}$, $\varepsilon_{MST}$, $\varepsilon_{MSB}$, and $\varepsilon_{MGB}$ denote the minimum of the vertical displacement at the top of the SMA layer, the maximum of the transverse strain at the top and the bottom of the SMA layer, and the maximum of the transverse strain at the bottom of the GA layer, respectively, and the location of these responses are considered to be the location of the critical response of the deck pavement. ${\text{U}}_{MST}$, $\varepsilon_{MST}$, $\varepsilon_{MSB}$, and $\varepsilon_{MGB}$ are listed in Table 2 under different interface condition, where the responses under the ICB condition are greater than them under the full-bond condition, especially $\varepsilon_{MST}$. This may imply that the full-bond condition underestimate the responses of the deck pavement comparing with the ICB condition, and the top-down cracking may easily be produced at the top of the SMA layer between the dual-tire under the high temperature. This also indicate that the interface bonding condition between the adjacent layers have a significant correlation with the responses prediction and also have a obvious influence on the assessment of service life of the deck pavement. Hence, the responses of the deck pavement will be estimated by the ICB condition and the ICB model at the high temperature in the following parts.

**Table 2:** Responses under different interface condition.

Bonding condition	${\text{U}}_{MST}$ (mm)	$\varepsilon_{MST}$ (10^−6^)	$\varepsilon_{MSB}$ (10^−6^)	$\varepsilon_{MGB}$ (10^−6^)
Full-bond condition	− 0.567999➀	319.998	219.211	118.349
ICB condition	− 0.691528➁	675.144	231.310	204.993
Ratio (((➁ − ➀)/➀) * 100%)	21.75%	110.98%	5.52%	73.21%

### Critical responses of the deck pavement with bonding failure

**Figure 11:**
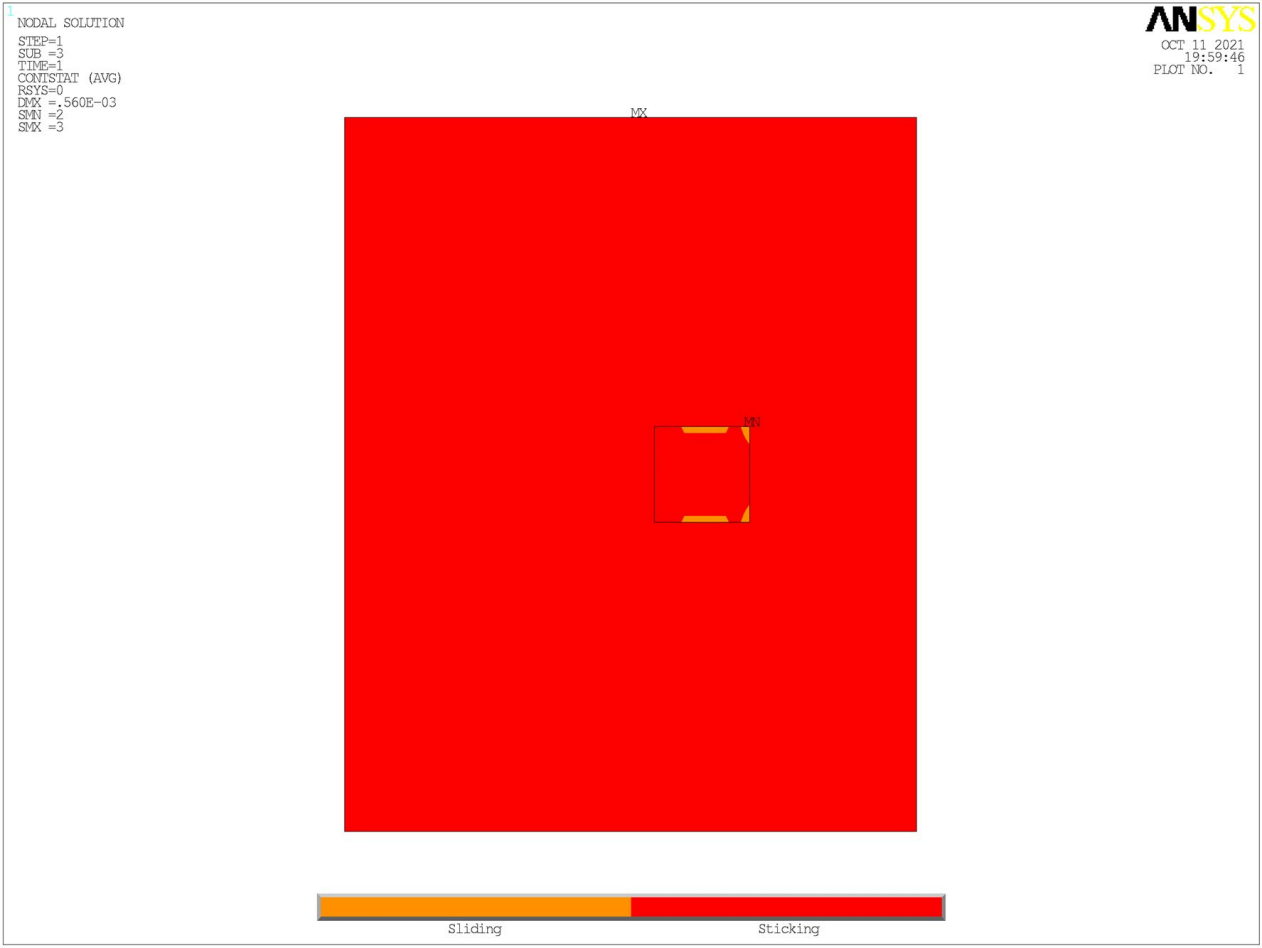
Contact bonding state between the GA layer and the steel plate.

The phenomenon of the bonding failure between the adjacent interface at somewhere may happen with the aging of the deck pavement and the repeatedly action of vehicle load. To simulate the phenomenon of the bonding failure and investigate the effect on the responses of the deck pavement, the bonding failure is considered between the GA layer and the steel plate. The six types of the disengaging area shown in Fig. 5 are considered to be the possible type of bonding failure between the adjacent layers. The critical responses of the deck pavement are still marked ${\text{U}}_{MST}$,$\varepsilon_{MST}$,$\varepsilon_{MSB}$, and $\varepsilon_{MGB}$, and they are calculated with different kind of the disengaging area at the high temperature and listed in Table 3. ${\text{U}}_{MST}$ and $\varepsilon_{MST}$ change a little bit from the type ‘T0’ to the type ‘T2’ when the disengaging area is less than the contact area of the single tire. On the contrary, ${\text{U}}_{MST}$ and $\varepsilon_{MST}$ increase gradually from the type ‘T3’ to the type ‘T5’ when the disengaging area is equal or greater than contact area of a single tire. From the ‘T0’ to the type ‘T5’, $\varepsilon_{MSB}$ is almost unchanged, but $\varepsilon_{MGB}$ change obviously. This indicate that the size of the disengaging area and it’s position affect the responses of the deck pavement. The responses at the top of the SMA layer increase slowly with the increase of the size of the disengaging area, but the responses at the bottom of the GA layer increase clearly. Besides, the contact bonding state under the type ‘T5’ is shown in Fig. 11, the red part means sticking and the yellow part means sliding. The maximum of sliding between the GA layer and the steel plate is 0.0394 mm, and no sliding occur under other type of the disengaging area. This denote that the sliding will produce with the increase of the disengaging area between the GA layer and the steel plate and have a trend to increase.

**Table 3:** Critical responses with interface bonding failure at the high temperature.

Type	${\text{U}}_{MST}$ (mm)	$\varepsilon_{MST}$ (10^−6^)	$\varepsilon_{MSB}$ (10^−6^)	$\varepsilon_{MGB}$ (10^−6^)
T0	− 0.692	675.144	231.310	204.993
T1	− 0.692	675.126	231.229	226.792
T2	− 0.693	674.738	230.641	324.183
T3	− 0.701	692.300	225.428	417.966
T4	− 0.713	723.817	221.061	464.786
T5	− 0.718	734.770	232.064	482.488

### Critical responses of deck pavement under moving loads

**Figure 12:**
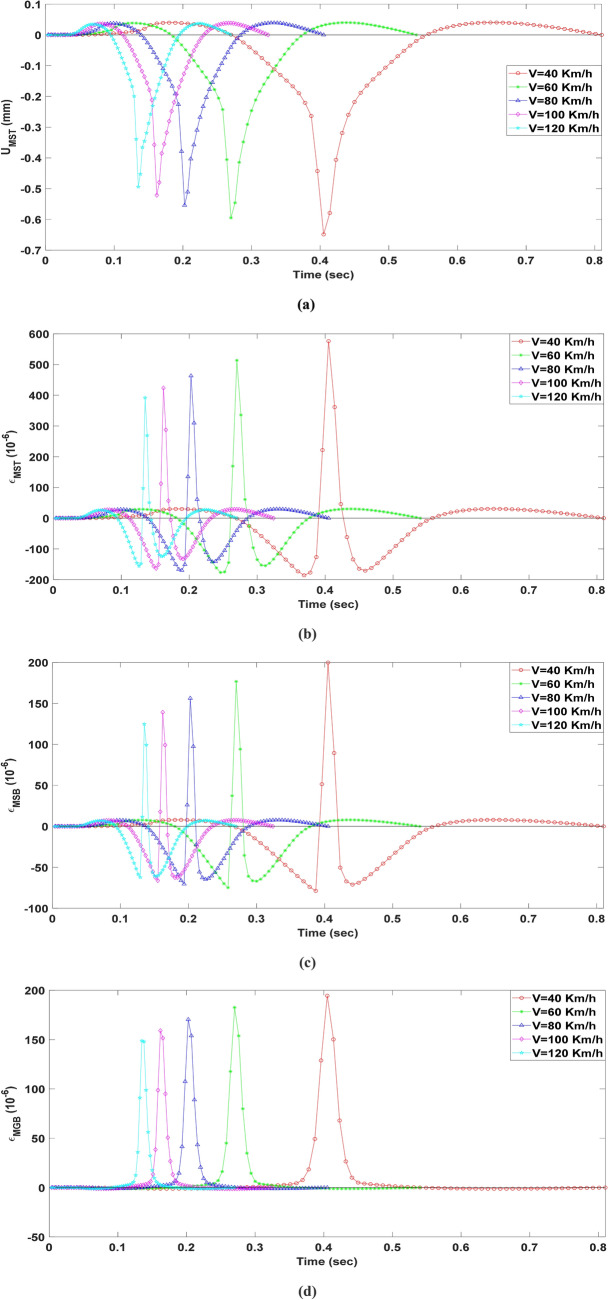
Critical responses of deck pavement under moving loads.

The response analysis under moving loads is a better way to study the behaviors of the deck pavement, so the critical response of deck pavement at different vehicle speed were calculated and shown in Fig. 12. The responses of ${\text{U}}_{MST}$, $\varepsilon_{MST}$ and $\varepsilon_{MSB}$ all have an alternating process of tension and compression, but $\varepsilon_{MGB}$ have an obvious process of tension. Although the vehicle speed is different, the trend of responses of the deck pavement are very similar. When the vehicle speed increase, the period of loads acted on the surface of the deck pavement decrease and the critical responses of the deck pavement also decrease. The local effect of the vehicle loads is very significant, when the vehicle loads close to the critical position. The peak value of critical responses with different vehicle speed at the high temperature are listed in Table 4. ${\text{U}}_{MST}$, $\varepsilon_{MST}$, $\varepsilon_{MSB}$, and $\varepsilon_{MGB}$ decrease 19.72%, 26.51%, 30.36% and 18.20%, respectively, when the vehicle speed increase form 40 to 120 km/h. This may imply that increasing the vehicle speed can reduce the strains of the deck pavement and the possibility of damage, but this is a potentially dangerous situation for safe driving.

**Table 4:** Peak value of critical responses with different vehicle speed at the high temperature.

Vehicle speed (Km/h)	${\text{U}}_{MST}$ (mm)	$\varepsilon_{MST}$ (10^−6^)	$\varepsilon_{MSB}$ (10^−6^)	$\varepsilon_{MGB}$ (10^−6^)
40	− 0.649➀	576.174	199.841	194.503
60	− 0.595	513.630	176.711	182.621
80	− 0.554	463.152	156.332	170.472
100	− 0.521	423.448	139.179	159.108
120	− 0.494➁	392.107	124.855	148.819
Ratio ((➁ − ➀)/➀ × 100%)	− 23.88%	− 31.95%	− 37.52%	− 23.49%

### Influence of the parameter of OSD on the critical responses of deck pavement

The thickness of the deck plate and the thickness of the U-rib have an influence on the responses of asphalt pavement, so sensitivity analysis of these parameters are performed to improve the design of OSD. The critical responses with the different thickness of deck plate at the high temperature are calculated and listed in Table 5, and the critical responses with the different thickness of U-rib at the high temperature are calculated and listed in Table 6.

**Table 5:** Critical responses with the different thickness of deck plate at the high temperature.

Thickness of deck plate (mm)	${\text{U}}_{MST}$ (mm)	$\varepsilon_{MST}$ (10^−6^)	$\varepsilon_{MSB}$ (10^−6^)	$\varepsilon_{MGB}$ (10^−6^)
12	− 0.740➀	826.949	220.949	247.263
14	− 0.692	675.144	231.310	204.993
16	− 0.654	577.535	237.876	175.814
18	− 0.625➁	517.940	242.775	156.120
Ratio ((➁ − ➀)/➀ × 100%)	− 15.57%	− 37.37%	9.88%	− 36.86%

**Table 6:** Critical responses with the different thickness of U-rib at the high temperature.

Thickness of U-rib (mm)	${\text{U}}_{MST}$ (mm)	$\varepsilon_{MST}$ (10^−6^)	$\varepsilon_{MSB}$ (10^−6^)	$\varepsilon_{MGB}$ (10^−6^)
6	− 0.796➀	600.545	193.559	205.181
8	− 0.692	675.144	231.310	204.993
10	− 0.626	718.416	251.410	204.095
12	− 0.580➁	744.542	262.164	202.801
Ratio ((➁ − ➀)/➀ × 100%)	− 27.19%	23.98%	35.44%	− 1.16%

In Table 5, with the increase of the thickness of deck plate from 14 to 18 mm, ${\text{U}}_{MST}$,  $\varepsilon_{MST}$ and $\varepsilon_{MGB}$ decrease 15.57%, 37.37% and 36.86%, respectively, but $\varepsilon_{MSB}$ increase 9.88%. It is clearly that the increase of the thickness of the deck plate will enhance the stiffness of the orthotropic steel deck bridges with asphalt pavement, and decrease the some responses of asphalt pavement so as to prevent the premature damage of deck pavement. On the other hand, the increase of the thickness for deck plate will inevitably lead to the increase of construction costs, so it is also undesirable. Considering the deformation of the deck pavement in Table 5 and the construction costs, the deck plate with the thickness of 14 mm may be a good choice, and the deck plate with the thickness of 16 mm may be more suitable for the condition of heavy traffic and long-term high temperature.

In Table 6, with the increase of the thickness of U-rib from 6 to 12 mm, ${\text{U}}_{MST}$ and $\varepsilon_{MGB}$ decrease 27.19% and 1.16%, respectively, but $\varepsilon_{MST}$ and $\varepsilon_{MSB}$ increase 23.98% and 35.44%, respectively. It means that the increase of the thickness of U-rib can reduce ${\text{U}}_{MST}$ of deck pavement, but $\varepsilon_{MST}$ and $\varepsilon_{MSB}$ of deck pavement all raise. So the increase of the thickness for U-rib will may promote the risk of cracking of both the bottom and the top of asphalt pavement. Considering saving costs and the deformation of deck pavement, the reasonable thickness of U-rib may be 8 mm by comparing the responses of deck pavement in Table 6.

## Conclusions

In order to capture the interface state between adjacent layers and obtain the credible responses of deck pavement under vehicle loads, a two-layer paving structure was investigated to estimate the response characteristics of the OSD pavement by finite element model at the high temperature, and some important results of this study are summarized as follows:

1. An interface contact bonding model was used to simulate the interface contact bonding condition between adjacent layers for the OSD pavement. Comparing the response results with previous researches, the response results from the interface contact bonding model are basely acceptable and credible.
2. The responses under the full-bond condition and the interface contact bonding condition were calculated, respectively, and it was found that there was a clear difference between the responses at the bottom of SMA layer and GA layer under the two kind of the interface condition. However, the responses with the interface contact bonding condition were more reasonable according to previous researches.
3. A clear advantage is that the interface contact bonding model can simulate the bonding failure at somewhere between adjacent layers. The bonding failure between the GA layer and the steel plate was investigated, which have an obvious effect on the transverse strains at the bottom of the GA layer with the increase of the disengaging area, and relatively weaker influence on the responses at the top of the SMA layer.
4. The responses of the deck pavement under moving loads were calculated, and the peak value of the responses of SMA layer and GA layer all decreased with the increase of the vehicle speed. Hence, restricting vehicle speed within a reasonable range can not only reduce the responses of the deck pavement, but also ensure driving safety.
5. By the sensitivity analysis of thickness parameters, it is advised that the thickness of the deck plate is no less than 14 mm, and the thickness of it is no less than 16 mm for the condition of heavy traffic and long-term high temperature. The 8 mm thickness of the U-rib is a reasonable and good choice.
